# Incidence and factors associated with being lost to follow-up among people living with HIV and receiving antiretroviral therapy in Nyarugenge the central business district of Kigali city, Rwanda

**DOI:** 10.1371/journal.pone.0275954

**Published:** 2022-10-13

**Authors:** Daniel Ntabanganyimana, Lawrence Rugema, Jared Omolo, Olivier Nsekuye, Samuel Sewava Malamba

**Affiliations:** 1 Rwanda Field Epidemiology and Laboratory Training Program, Department of Epidemiology and Biostatistics, University of Rwanda, Kigali, Rwanda; 2 Ministry of Health, Kigali, Rwanda; 3 University of Rwanda/School of Public Health, Kigali, Rwanda; 4 CGH DGHT, Centers for Disease Control and Prevention, Kigali, Rwanda; 5 Rwanda Biomedical Centre, Public Health Surveillance & Emergency Preparedness and Response, Kigali, Rwanda; Kasetsart University, THAILAND

## Abstract

**Background:**

Lost to follow-up (LTFUP) continues to threaten the sustainability of antiretroviral therapy (ART) benefits and success of ART programs. We determined the incidence and predictors of LTFUP among people living with HIV (PLHIV) on ART in Nyarugenge the Central Business District of Kigali city.

**Methods:**

A cohort of PLHIV who initiated ART in 2018 was retrospectively studied for 24 months. Using health facility records, a person who had no record of contact with the health facility for at least three consecutive months was considered LTFUP. LTFUP incidence rates were computed, and the Fine-Gray’s competing risk regression models were used to determine factors associated with time to first LTFUP. Generalized estimating equations (GEEs) were used to analyze repeated measurement outcomes of LTFUP and predictors of LTFUP.

**Results:**

Of 950 participants, 581 (61.2%) were females and 866 (91.2%) were 15 to 49 years old. From 1,586.1 person years of observation (pyo), 148 participants got LTFUP for 451 times. The incidence rate to first event was 9.4 per 100 pyo (95%CI:7.9–10.9) and 31.8 per 100 pyo (95%CI:29.0–34.4) to multiple events. WHO stage, marital status, employment status and person to contact when PLHIV is not reachable were associated with time to first LTFUP event. However, an average participant with a contact person who was not a Community Health Worker (CHW) or a peer educator had higher incidence of LTUP (aIRR = 2.69, 95%CI: 1.43–5.06), an average single patients had higher incidence of LTFUP (aIRR = 1.74, 95%CI: 1.28–2.34) compared to married/co-habiting, and an average self-employed had higher incidence of LTFUP (aIRR = 1.51, 95%CI: 1.14–2.01) compared to participants employed by others. Furthermore, an average PLHIV living out-of-the health facility catchment area had higher incidence of LTFUP (aIRR = 1.55, 95%CI: 1.19–2.01) compared to an average PLHIV living in the health facility catchment area whereas, an average children initiated on first line had lower incidence of LTUP (aIRR = 0.43, 95%CI: 0.21–0.86) compared to adults.

**Conclusion:**

Using CHW and peer educators as contact persons can help to reduce LTFUP while, targeted sensitization and service delivery are needed for single, self-employed and, patients living out of the health facility catchment area.

## Background

The human immunodeficiency virus (HIV) continues to be a public health issue worldwide. By the end of 2020, the World Health Organization (WHO) estimated 37.7 million people living with HIV (PLHIV) and 36.3 million lives claimed by HIV. Over two-thirds (about 25.4 million) of all PLHIV were living in the African region of the WHO. However, the increased availability of effective prevention, diagnosis, treatment, and care has made HIV a manageable lifelong disease. Thus, allowing PLHIV to live healthy for many years. Response efforts from countries steadily increased the coverage of HIV services. By the end of 2020, the coverage of ART was 73% whereas by June 2021, 28.2 million PLHIV were receiving antiretroviral therapy (ART) worldwide. Due to ART, about 15.3 million lives were saved and new HIV infections fell by 39% between 2000 and 2019 [1].

The Rwanda population-based HIV impact assessment (RPHIA 2018–2019) reported an HIV prevalence which has stabilized at 3% among adults in Rwanda. This implies approximately 210,200 adults PLHIV (10–64 years) nationally. Adults represent 97.5% of all PLHIV in Rwanda [2].

Based on the evidence from clinical trials on the benefits of ART to patients and the reduced capacity of transmitting HIV to non-infected partners, WHO, in the guidelines of 2016, launched a new policy recommendation that raised the bar to treat all PLHIV. With this recommendation, it was expected that there would be an increase of patients on ART while new infections reduce. However, the WHO has noted that this is only achievable if the improvement is also made in retaining patients on ART [3–5].

In most settings, regular visits at the health facilities (HF) are critical to have complete care for HIV patients. It is during these appointments that health care providers examine patients for clinical progress, provide ART and counseling to minimize HIV transmission in susceptible populations [6]. Even with the importance of regular attendance at health facilities, cohort studies have demonstrated that lost to follow-up (LTFUP) in ART programs is the main cause of attrition followed by death in Sub–Saharan Africa [7].

Rwanda launched the ‘treat all’ strategy in July 2016 [4, 8]. With this strategy, each individual confirmed HIV positive initiates ART regardless of any other criteria, immunological and/or clinical. Since the rollout of the ‘treat all’ strategy in Rwanda, the number of people living with HIV (PLHIV) on ART has increased. As of June 2021, 207,089 PLHIV were on ART, representing 92.5% of all PLHIV in Rwanda [9]. In earlier years, Kigali city contributed more people on ART than any other province in Rwanda [10]. Since the initiation of ‘treat all’ strategy in Rwanda, increased levels of viral load (VL) monitoring/coverage and suppression have been observed [8].

Although total adherence to ART is beneficial and preferred, it gets more difficult and complicated to retain in care or to have all PLHIV to fully adhere to ART for life. Therefore, sometimes some PLHIV get LTFUP [11]. Yet, patients who become LTFUP are susceptible to poor health outcomes including failure to suppress VL and developing drug resistance among others. Moreover, as measures for prevention and reduction of LTFUP would require a thorough understanding of its characteristics. However, little is known about the incidence of lost to follow up among PLHIV receiving ART since there are a few studies in Rwanda, which have documented the levels of retention in care and adherence to ART.

This study was conducted to estimate the incidence of LTFUP among PLHIV receiving ART and to determine the factors associated with being LTFUP.

## Methods

### Study design

This is a retrospective cohort study conducted among PLHIV who initiated ART between January 2018 and December 2018.

### Study site and settings

This study was conducted in the health facilities of Nyarugenge District in the city of Kigali. Nyarugenge is one of the three districts which constitutes Kigali city, the largest and capital city of Rwanda. Geographically, the City of Kigali is located in the center of the country and shares borders with three (North, South and East) of the four provinces of Rwanda. Approximately 1.2 million people inhabit Kigali. About 60% of this population is young and 50% are female. According to the 2018–2019 RPHIA, the prevalence of HIV in Kigali is 4.3%, higher than any of the other provinces, whose prevalence varies between 2.2% and 3.0%. Kigali city is made-up of three districts, which included Gasabo, Kicukiro and Nyarugenge. Nyarugenge district is the heart of Kigali city and contains most of the city’s businesses.

### Study participants

This study included all PLHIV who initiated ART between January and December 2018 among public health facilities of Nyarugenge District within the City of Kigali.

### Variables, data sources and measurement

Participants’ data were abstracted from health facility records (Secondary data). These included ART linkage register, patient charts/computer tools used in routine data collection at health facilities for clinical monitoring and follow up of PLHIV and were used as data sources. The outcome variable was defined as being lost to follow up from the ART care services since initiation of treatment. A PLHIV was considered lost to follow up when he or she had missed contact with the health facility for a period of ninety consecutive days since her or his last visit to the clinic within a period of two years after initiation of ART. At the time of the study, a person on ART was given 30 days to refill and for clinical review. Thus ninety days would mean missing three health facility visits and refills. On each scheduled health facility visit, the ART nurse established the reason for missing a scheduled visit from the contact person when the PLHIV was not reachable. These included, refused to continue taking ART/participation, stopped taking ART due to side effects, reported dead, not able to be reached using all the available contacts. Multiple or repeated events of LTFUP were considered when participants experienced new or recurrent episodes of LTFUP after returning to care from the first event of LTFUP. The following categories of data were collected:

Socio demographic variables (age, sex, place of residence, profession, marital status and having a fixed home). Clinical variables: Mode of admission (HIV Testing and Counselling (HTC), Provider Initiated Test (PIT), Prevention from Mother to Child Transmission (PMTCT) and Tuberculosis service (TB), WHO clinical stage, weight, height to calculate Body Mass Index (BMI), HIV and tuberculosis co-infection; Biological variables -CD4 counts, Viral Load (VL), medication data variables—(ART combinations), and drug adherence variables (stability of HIV patient vis-a-vis attending scheduled visits (ART refill, biological and clinical follow up visits) and time of being LTFUP were collected.

### Data collection tools and process

An electronic data abstraction tool accessible on computer was designed in Microsoft Excel and used to facilitate the data collection. The Excel data collection tool was built using drop down menu selection and data formatting where applicable to avoid typing errors and changes in date formats between computes. Data collection team constituted of HIV mentors from Muhima District Hospital. At the health facility, the data collection team worked with the data manager and the nurse in charge of the ART clinic to review the health facility records, abstract and enter data electronically for this study.

### Data management and analysis

Data cleaning, checking for inconsistencies and completeness was conducted in Microsoft Excel. Statistical data analysis was conducted using Stata Version 13 (Statacorp LP, College Station, Texas 77845 USA). Participants’ characteristics were described in terms of percentages. The incidence rates of first time to being LTFUP computed after declaring data to be survival analysis and expressed per 100 person years of follow-up. Follow-up time started at day of ART initiation and was censored at the end of 24 months of follow-up or when a patient was transferred out or when reported dead. Fine-Gray’s competing risk regression models with death as a competing risk for LTFUP were used to determine factors associated with time to first LTFUP event. All variables with p<0.1 at the univariate level were included in the multi-variable model and using a backward elimination method, only those variables with p<0.05 were kept in the final model. Generalized estimating equations with the Poisson family and a GEE-exchangeable correlation matrix were used to analyze repeated measurement outcomes of LTFUP and predictors of LTFUP. Since we used the actual follow-up time rather than the interval for the time variable, a sensitivity analysis, using a random effect Poisson model was also fitted. First time LTFUP incidence rate and repeated measurement outcomes of LTFUP 95% confidence intervals (CI) were calculated.

### Ethical considerations and review

Ethical approval and clearance were obtained from the University of Rwanda, College of Medicine, and Health Sciences Institutional Review Board (Approval Notice: N0 152/CMHS IRB/2021). In addition, clearance to access the health facility records was guaranteed by the National Health Research committee of the Ministry of Health in Rwanda (Ref: NHR/2021/PROT/020). The need for consent was waived by the University of Rwanda, College of Medicine, and Health Sciences Institutional Review Board since this was a retrospective cohort study design posing minimal risk to minors or to their parents/guardians and the study team did not interact with human subjects or have access to personally identifiable information.

## Results

### Sociodemographic and clinical characteristics of PLHIV

In total, 950 PLHIV initiated ART in 12 health facilities located in Nyarugenge district from January to December 2018. All of them were included in this study. The majority of participants, 866 (91.2%) were aged between 15 to 49 years whereas 581 (61.2%) were females. Four hundred and thirty-five (45.8%) participants were married or cohabitating while 323 (34.0%) were single. Moreover, 448 (47.2%) were self-employed and almost a half of the participants (478) were living in the health facility catchment area. Regarding person of contact when PLHIV is not reachable or missed an appointment, 708 (74.5%) participants provided a family member (Table 1).

**Table 1:** Distribution of sociodemographic and clinical characteristics of PLHIV and Lost to follow up among PLHIV who initiated ART in 2018, Nyarugenge district, Rwanda.

Characteristics	N = 950 (Column %)	Number with 1^st^ LTFUP N = 148 (Row %)
**Sex**		
Female	581 (61.2)	95 (16.4)
Male	369 (38.8)	53 (14.4)
**Age group**		
< 15 years	32 (3.4)	5 (15.6)
15–24	190 (20.0)	40 (21.1)
25–29	150 (15.8)	28 (18.7)
30–39	351 (36.9)	48 (13.7)
40–49	175 (18.4)	19 (10.9)
50 +	52 (5.5)	8 (15.4)
**Marital status**		
Married/cohabitating	435 (45.8)	49 (11.3)
Divorced/Separated/Widow	192 (20.2)	28 (14.6)
Single	323 (34.0)	71 (22.0)
**Employment status**		
Employed	369 (38.8)	37 (10.0)
Self employed	448 (47.2)	87 (19.4)
Unemployed	133 (14.0)	24 (18.1)
**Means of transport**		
Moto/bicycle/Own car	60 (6.3)	6 (10.0)
Public transport	856 (90.1)	137 (16.0)
Missing	34 (3.6)	5 (14.7)
**BMI at ART initiation**		
Underweight	135 (14.2)	20 (14.8)
Normal weight	584 (61.5)	96 (16.4)
Overweight	178 (18.7)	28 (15.7)
Missing	53 (5.6)	4 (7.6)
**PLHIV has a fixed home**		
No	591 (62.2)	86 (14.6)
Yes	359 (37.8)	62 (17.3)
**Person of contact when PLHIV is not reachable**		
CHW/Peer Educator	109 (11.5)	4 (3.7)
Family member	708 (74.5)	117 (16.5)
Other/None	133 (14.0)	27 (20.3)
**Number of HH members**		
≤ 3 HH members	702 (73.9)	111 (15.8)
> 3 HH members	248 (26.1)	37 (14.9)
**PLHIV residence place**		
In the catchment area of the HF	472 (49.7)	70 (14.8)
Out of the catchment area of the HF	478 (50.3)	78 (16.3)
**TB Co- infection**		
No	900 (94.7)	143 (15.9)
Yes	21 (2.2)	2 (9.5)
Missing	29 (3.1)	3 (10.3)
**Mode of admission**		
HTC	642 (67.6)	105 (16.4)
PIT	140 (14.7)	23 (16.4)
PMTCT	105 (11.1)	13 (12.4)
TB service	9 (1.0)	2 (22.2)
Missing	54 (5.7)	5 (9.3)
**Who stage**		
I	734 (77.3)	108 (14.7)
II	160 (16.8)	34 (21.3)
III&IV	56 (5.9)	6 (10.7)
**ART regimen**		
Adult 1st Line/Adult alternative 1st line	900 (94.7)	144 (16.0)
Children 1st line	50 (5.3)	4 (8.0)
**CD4 count at Initiation**		
≤ 300	326 (34.3)	55 (16.9)
> 300	624 (65.7)	93 (14.9)
**Viral load at 18 months**		
Undetectable VL	535 (56.3)	6 (1.2)
Detectable VL	32 (3.4)	1 (3.2)
Missing	383 (40.3)	141 (36.9)
**Level of stability**		
Unstable	551 (58.0)	138 (25.1)
Stable A	277 (29.2)	1 (0.4)
Stable B	95 (10.0)	4 (4.3)
Missing	27 (2.8)	5 (18.6)

### Incidence rate of lost to follow up among PLHIV who initiated ART in 2018, Nyarugenge district, Rwanda

The cohort of 950 PLHIV was followed for 1,586.1 person years. The number of participants who were LTFU and never come back as by the end of follow-up was 118; those who were considered LTFU but returned in care was 30; those reported dead were 6; and those transferred out was 128. The average number of LTFU events per person among those who had ever been LTFU was 3.0 (451 LTFU events/148 persons LTFU). The overall incidence rate to first LTFUP event was 9.4 per 100 pyo (95%CI:7.9–10.9) and 31.8 per 100 pyo (95%CI:29.0–34.4) to multiple events of LTFUP (Table 2).

**Table 2:** Using Fine-Gray’s competing risk regression models to determine factors associated with time to first LTFUP event among PLHIV who initiated ART in 2018, Nyarugenge district, Kigali Rwanda.

Variables	Total pyo to 1st LTFUP	Number of 1^st^ LTFUP events	Incidence Rate to 1^st^ LTFUP/100 person-years	95% CI	Crude sub- HRs	95% CI	Wald’s test p-value	Adjusted sub- HRs	95% CI	p-Value
**Sex**							0.351			
Female	960	95	9.90	(8.09–12.10)	1.00					
Male	626	53	8.46	(6.46–11.08)	0.85	(0.61–1.19)				
**Age group**							0.065			
< 15 years	60	6	10.04	(4.50–22.34)	1.00					
15–24	298	39	13.09	(9.56–17.92)	1.52	(0.60–3.84)				
25–29	249	28	11.23	(7.75–16.26)	1.28	(0.49–3.31)				
30–39	581	48	8.26	(6.22–10.96)	0.94	(0.37–2.36)				
40–49	307	19	6.20	(3.95–9.71)	0.70	(0.26–1.88)				
50 +	92	8	8.71	(4.36–17.41)	0.98	(0.32–3.00)				
**Marital status**							<0.001			
Married/Cohabitating	752	49	6.51	(4.92–8.62)	1.00			1.00		
Divorced/Separated/Widowed	324	28	8.65	(5.96–12.52)	1.31	(0.82–2.08)		1.35	(0.84–2.16)	0.214
Single	510	71	13.92	(11.03–17.56)	2.15	(1.50–3.08)		2.10	(1.44–3.07)	**<0.001**
**Employment status**							0.0014			
Employed	630	37	5.87	(4.25–8.10)	1.00			1.00		
Self employed	731	87	11.90	(9.65–14.68)	2.02	(1.38–2.96)		1.89	(1.29–2.76)	**0.001**
Unemployed	225	24	10.67	(7.14–15.91)	1.80	(1.07–3.01)		1.37	(0.79–2.37)	0.259
**Means of transport**							0.453			
Moto/Bicycle/Own car	102	6	5.86	(2.63–13.04)	1.00					
Public transport	1,423	137	9.63	(8.14–11.38)	1.67	(0.74–3.77)				
Missing	60	5	8.28	(3.44–19.87)	1.43	(0.44–4.63)				
**BMI at ART initiation**							0.318			
Underweight	226	20	8.87	(5.71–13.74)	1.00					
Normal weight	958	96	10.02	(8.20–12.23)	1.14	(0.70–1.85)				
Overweight	303	28	9.24	(6.37–13.38)	1.03	(0.58–1.83)				
Missing	99	4	4.02	(1.50–10.72)	0.45	(0.16–1.31)				
**PLHIV Has a fixed home**							0.232			
No	998	86	8.62	(6.97–10.64)	0.82	(0.59–1.14)				
Yes	588	62	10.54	(8.21–13.51)	1.00					
**Person of contact when PLHIV is not reachable**							0.004			
CHW/Peer Educator	191	4	2.10	(0.78–5.59)	1.00			1.00		
Family member	1,179	117	9.93	(8.28–11.89)	4.71	(1.76–12.62)		4.56	(1.69–12.30)	**0.003**
Other/None	217	27	12.45	(8.53–18.14)	5.92	(2.09–16.77)		5.67	(1.98–16.22)	**0.001**
**Number of HH members**							0.565			
≤ 3 HH members	1,157	111	9.60	(7.97–11.56)	1.00					
> 3 HH members	430	37	8.61	(6.24–11.88)	0.90	(0.62–1.30)				
**PLHIV residence place**							0.235			
In the HF catchment area	825	70	8.48	(6.71–10.72)	1.00					
Out of the HF catchment area	761	78	10.25	(8.21–12.79)	1.21	(0.88–1.67)				
**TB Co- infection**							0.471			
No	1,496	143	9.56	(8.11–11.26)	1.00					
Yes	38	2	5.24	(1.31–20.95)	0.55	(0.14–2.19)				
Missing	52	3	5.72	(1.84–17.74)	0.60	(0.19–1.84)				
**Mode of admission**							0.530			
HTC	1,069	105	9.83	(8.11–11.89)	1.00					
PIT	225	23	10.22	(6.78–15.37)	1.05	(0.67–1.64)				
PMTCT	176	13	7.38	(4.28–12.71)	0.76	(0.42–1.36)				
TB service	17	2	12.06	(3.01–48.22)	1.23	(0.32–4.76)				
Missing	100	5	5.02	(2.09–12.05)	0.51	(0.21–1.27)				
**WHO stage**							0.084			
I	1,231	108	8.77	(7.26–10.58)	1.00			1.00		
II	261	34	13.01	(9.29–18.20)	1.47	(1.00–2.17)		1.44	(0.98–2.11)	**0.064**
III&IV	94	6	6.41	(2.88–14.27)	0.72	(0.32–1.63)		0.66	(0.28–1.54)	0.333
**ART regimen initiated**							0.172			
Adult 1st Line/Adult alternative 1st line	1,501	144	9.60	(8.15–11.29)	1.00					
Children 1st line	85	4	4.68	(1.75–12.47)	0.49	(0.18–1.36)				
**CD4 count at Initiation**							0.512			
≤ 300	546	55	10.08	(7.74–13.13)	1.12	(0.80–1.56)				
> 300	1,041	93	8.94	(7.28–10.94)	1.00					

### Factors associated with first LTFUP event among PLHIV who initiated ART 2018, Nyarugenge district, Rwanda

Being single, being self-employed, not having a CHW or Peer educator as a person of contact or not having any person of contact when not reachable and being on WHO stage II were significantly associated with first lost to follow-up event.

Participants who self-reported being single were two times as likely to be at risk of the first LTFUP event (adjusted sub-HRs = 2.10, 95%CI = 1.44–3.07) compared to married or cohabitating PLHIV. Being self-employed was associated with high risk of the first LTFUP event (adjusted sub-HRs = 1.89, 95% CI: 1.29–2.76) compared to PLHIV employed by others. The risk of first LTFUP event was very high (adjusted sub-HRs: 5.92, 95%CI = 2.09–16.77) in PLHIV who did not have a person to contact or did not have either a CHW or peer educator (Table 2).

### Factors associated with—LTFUP events among PLHIV who initiated ART in 2018, Nyarugenge district, Rwanda after adjusting for repeated outcome measures

Marital status, employment status, person of contact when PLHIV is not reachable, PLHIV residing in or outside the catchment area of the health facility, and age of PLHIV were significantly associated with being lost to follow-up -.

An average single PLHIV—had higher incidence of LTFUP (aIRRs = 1.74, 95% CI: 1.28–2.34) compared to an average married PLHIV. An average self-employed PLHIV had higher incidence of LTFUP (aIRRs = 1.51, 95% CI: 1.14–2.01) compared to an average PLHIV employed by others. An average PLHIV who does not have a CHW or a peer educator as the person of contact or who does not have any person of contact had higher incidence of LTFUP (aIRRs = 2.69, 95% CI: 1.43–5.06) compared to an average PLHIV who had a CHW or peer educator as a person of contact. Furthermore, an average PLHIV who was leaving outside the catchment area of the health facility had higher incidence of LTFUP (aIRRs = 1.55, 95% CI: 1.19–2.01) compared to an average PLHIV leaving in the catchment area of the health facility. However, an average child living with HIV had lower incidence of LTFUP (aIRRs = 0.43, 95% CI: 0.21–0.86) compared to an average adult PLHIV (Table 3).

**Table 3:** Generalized estimating equations analyzing repeated measurement outcomes of LTFUP and associated factors among PLHIV who initiated ART in 2018, Nyarugenge district, Rwanda.

Variables	Total pyo to 1st LTFUP	Number of times LTFUP	Crude IRRs	95% CI	p-value	Adjusted IRRs	95% CI	P-Value
**Sex**				** **				
Female	960	371	1.00					
Male	626	180	0.81	(0.62–1.06)	0.122			
**Age group (years)**								
< 15	60	21	1.00					
15–24	298	165	1.52	(0.62–3.68)	0.358			
25–29	249	104	1.11	(0.45–2.75)	0.822			
30–39	581	168	0.97	(0.41–2.34)	0.953			
40–49	307	63	0.69	(0.27–1.73)	0.422			
50 +	92	30	0.87	(0.31–2.48)	0.798			
**Marital status**								
Married/cohabitating	752	193	1.00			1.00		
Divorced/Separated/Widowed	324	113	1.27	(0.88–1.84)	0.205	1.39	(0.96–2.00)	0.078
Single	510	245	1.68	(1.26–2.23)	**<0.001**	1.74	(1.28–2.34)	**<0.001**
**Employment status**								
Employed	630	142	1.00			1.00		
Self employed	731	327	1.59	(1.20–2.12)	**0.001**	1.51	(1.14–2.01)	**0.004**
Unemployed	225	82	1.32	(0.87–1.99)	0.195	1.09	(0.70–1.69)	0.703
**Means of transport**								
Moto/Bicycle/Own car	102	17	1.00					
Public transport	1,423	518	1.72	(0.99–3.00)	0.055			
Missing	60	16	1.21	(0.50–2.95)	0.674			
**BMI at ART initiation**								
Underweight	226	75	1.00					
Normal weight	958	355	1.11	(0.76–1.62)	0.590			
Overweight & Obese	303	104	1.05	(0.67–1.66)	0.830			
Missing	99	17	0.48	(0.21–1.12)	0.090			
**PLHIV Has a fixed home**								
No	998	326	1.00					
Yes	588	225	1.11	(0.85–1.44)	0.445			
**Person of contact when PLHIV is not reachable**								
CHW/Peer Educator	191	16	1.00			1.00		
Family member	1,179	437	2.58	(1.48–4.52)	**0.001**	2.74	(1.57–4.78)	**<0.001**
Other/None	217	98	2.89	(1.54–5.42)	**0.001**	2.69	(1.43–5.06)	**0.002**
**Number of HH members**								
≤ 3 HH members	1,157	411	1.00					
> 3 HH members	430	140	0.89	(0.65–1.22)	0.472			
**PLHIV residence place**								
In the catchment area of the HF	825	305	1.00			1.00		
Out of the catchment area of the HF	761	246	1.45	(1.12–1.88)	**0.005**	1.55	(1.19–2.01)	**0.001**
**TB/HIV Co- infection**								
No	1,496	534	1.00					
Yes	38	5	0.33	(0.11–0.97)	**0.045**			
Missing	52	12	0.60	(0.25–1.44)	0.254			
**Mode of admission**								
HTC	1,069	390	1.00					
PIT	225	84	1.09	(0.76–1.56)	0.628			
PMTCT	176	53	0.91	(0.59–1.41)	0.680			
TB service	17	3	0.43	(0.12–1.60)	0.209			
Missing	100	21	0.54	(0.25–1.15)	0.110			
**WHO stage**								
I	1,231	402	1.00					
II	261	129	1.36	(0.99–1.89)	0.060			
III & IV	94	20	0.69	(0.37–1.26)	0.225			
**ART regimen initiated**								
Adult 1st Line/Alternative 1st Line	1,501	538	1.00			1.00		
Children 1st line	85	13	0.52	(0.26–1.06)	0.071	0.43	(0.21–0.86)	**0.017**
**CD4 count at Initiation**								
≤ 300	546	200	1.02	(0.77–1.34)	0.911			
> 300	1,041	351	1.00					

NB: A random effects Poisson regression model analyzing repeated measurement outcomes of LTFUP was fitted and the results obtained were similar to those obtained using GEE after adjusting for repeated outcome measures.

## Discussion

Currently, ARVs are being used in the whole cycle of HIV disease control, from HIV prevention to HIV treatment. Unfortunately, discontinuation of ART remains a big threat to its effectiveness, leading to higher HIV transmission rates, and to negative health outcomes.

This study reports high incidence rates of LTFUP among PLHIV on ART in Nyarugenge district. The overall incidence rate to first LTFUP event was 9.4 per 100 pyo (7.8 per 1000 person-months) which is slightly lower than the 8.8 per 1000 person-months found by a study conducted in an ART clinic in Mizan-Aman General Hospital, Ethiopia [12], however, much lower than the one found by another study conducted in Wakiso district of Uganda which found the incidence rate of LTFUP to be 21 per 1,000 pmo [13]. This difference could be explained by the difference in the population settings of the two studies. In addition, the incidence rate to multiple LTFUP events was 31.8 per 100 pyo (95%CI:29.0–34.4). High incidence rate of LTFUP in urban areas like Nyarugenge district is not an isolated observation but seems to be a common observation in other similar studies [14, 15]. The fact that the majority of PLHIV were running their own businesses could have made it difficult for them to attend all follow-up appointments as required. Similar to many other studies, the cumulative incidence of LTFUP increases with increasing time of follow-up [14, 15].

This study did not find any difference in LFTUP between gender as it was suggested by some other studies whereby, male PLHIV were shown to be at a higher risk of being LTFUP than females [12, 16, 17]. This may have been due to differences in sample populations used and therefore a fewer number of participants included in this study. Another possible reason could have been the difference in study settings, whereby Nyarugenge is the central business district of the city of Kigali and most people, males and females, are involved in running businesses, thus their behavior may have been more similar for both males and females than in other studies.

This study did not find significant association between LTFUP and TB-HIV coinfection, BMI, and CD4 counts at the initiation of ART as it was suggested by several other studies [11, 14–16, 18]. This difference may have arisen as a result of having insufficient power to detect the differences in this study and the difference in analysis conducted.

Marital status was found to be associated with LTFUP, whereby, single PLHIV were found to be two times more likely to be at risk of being LTFUP at least once. This implies that having a partner is protective against being lost to follow-up as reported by a study conducted in South Africa. Consistent to this finding, a study conducted in central Kenya reported that factors associated with LTFUP included being single or divorced [17, 19].

Being self-employed was found to be associated with being LTFUP. PLHIV who were self-employed were almost two times more likely to be at risk of being LTFUP at least once in two years following ART initiation.

Having a CHW or peer educator as a person of contact when the PLHIV is not reachable was significantly associated with a lower risk of being LTFUP as compared to not having one of them as your person of contact. This suggests that CHWs and peer educators play a big role in reminding PLHIV of their appointments considering that they live with the PLHIV in the community and most of the times they are neighbors. This is very important even when clinicians want to remind PLHIV of their appointments and their telephones are offline or do not have any telephone. The role of telephone calls in reminding PLHIV of their appointments has been described by other studies [12, 15, 20].

### Study limitations

Only Nyarugenge district data was analyzed and may not be generalizable to the whole City of Kigali or to the whole country. Secondly, the covariate data available for analysis was only those routinely corrected in the program, this might have limited our ability to analyze data on other potential factors that may influence LTFUP. The LTFUP definition limitation namely “a person who had no record of contact with the health facility for at least three consecutive months” may inadvertently misclassify those missing health facility visits, or those reported as self-transferred to other health facilities as being LTFUP. The issue of competing risk–some of the patients classified as LTFUP may have died, but given the short follow-up time, these would not be many, and we believe they may not have had a major effect on the magnitude or the general direction of study findings. Data quality issues associated with routinely collected program data including missing and erroneous data. The age of the analyzed data was from January to December of 2018 and may not reflect the current situation where differentiated service delivery allows stable PLHIV populations to benefit from multi-month-dispensing. However, the information derived from this analysis is still relevant in informing HIV programs on how to manage attrition in patients initiated on ART.

## Conclusion

This study revealed that PLHIV on ART who are single, self-employed, living outside the health facility catchment area and having a contact person who is not a CHW, or a peer educator were potentially at a higher risk of being LTFUP. Using CHW and peer educators as contact persons, can help to reduce LTFUP in PLHIV on ART while targeted sensitization and service delivery are needed for single, self-employed patients and those living out of the health facility catchment area. Moreover, multi month dispensing of ART approach would provide uninterrupted access to medication to busier PLHIV and improve the retention in care.
